# Efferocytosis and inflammation: a bibliometric and systematic analysis

**DOI:** 10.3389/fmed.2025.1498503

**Published:** 2025-02-10

**Authors:** Xin Cao, Fen Li, Xi Xie, Guanghui Ling, Xiaoyu Tang, Wenfang He, Jing Tian, Yan Ge

**Affiliations:** ^1^Department of Rheumatology & Immunology, The Second Xiangya Hospital, Central South University, Changsha, China; ^2^Clinical Medical Research Center for Systemic Autoimmune Diseases, Changsha, China; ^3^Department of Critical Care Medicine, The Second Xiangya Hospital, Central South University, Changsha, China

**Keywords:** efferocytosis, inflammation, bibliometrics, molecular mechanism, mesenchymal stem cells, nontumorous inflammatory diseases, cancer

## Abstract

**Objective:**

To visualize and analyze the trends and hotspots of efferocytosis and inflammation via bibliometric methods.

**Methods:**

Relevant articles and reviews from 2006 to 2023 were retrieved from the Web of Science Core Collection. The data were processed with CiteSpace, and some graphs were generated with Microsoft Excel (version 2016), VOSviewer, Scimago Graphica, Bibliometrix and R Studio.

**Results:**

A total of 1,003 papers were included, revealing a significant upward trend in efferocytosis and inflammation research. The United States (456, 45.46%), China (164, 16.35%) and the United Kingdom (99, 9.87%) were the three countries with the highest numbers of publications. Harvard University (84, 6.74%) contributes the most out of the top 5 institutions. Among the researchers in this field, Serhan CN was the author with the highest number of articles in the field (35, 3.49%), and deCathelineau AM first named “efferocytosis” in 2003. Keyword analysis identified “activation,” “tam receptors,” “docosahexaenoic acid” “systemic lupus erythematosus,” “myocardial infarction” and “alveolar macrophages” as core topics, indicating a concentrated trend in the mechanism of physiological state and inflammatory diseases such as autoimmune, cardiovascular, and pulmonary diseases. The latest surge words “inflammation resolution” and “cancer” in the keyword heatmap indicate future research directions.

**Conclusion:**

Research on the association between efferocytosis and inflammation has been a promising field. Key areas of focus include the crucial role of efferocytosis on tissue homeostasis and the pathogenesis of nontumorous inflammatory diseases. Future research will likely continue to explore these frontiers, with an emphasis on understanding efferocytosis in the context of chronic diseases and cancer, as well as developing novel therapeutic strategies.

## 1 Introduction

Billions of cells die every day in the human body (1). Efferocytosis, the process by which dying or dead cells are cleared by phagocytes, is crucial for maintaining tissue homeostasis and preventing inflammation. Professional phagocytes, such as macrophages and dendritic cells, are well-equipped with specific receptors and signaling pathways that facilitate the recognition and engulfment of apoptotic cells. Non-professional phagocytes, including epithelial cells and fibroblasts, can also participate in efferocytosis, though their mechanisms are less specialized (72). Efferocytosis is a cooperative process between phagocytes and apoptotic cells, regulated by signaling molecules known as “find-me” and “eat-me” signals. Phagocytes express receptors that recognize apoptotic ligands and interact with the cytoskeleton to bind to them, inducing phagosomes-lysosome fusion to degrade apoptotic cells (2). As apoptotic cells are phagocytosed, macrophages inhibit the production of inflammatory factors and mediate the repair process (3).

When efferocytosis is impaired, many apoptotic cells cannot be removed promptly and can accumulate in the body. This process is followed by secondary necrosis, rupture of cell membranes and the release of cellular contents, such as damage-associated molecular patterns (DAMPs) (4). Release of cellular contents triggers inflammation and the immune response, and leads to chronic inflammatory diseases and autoimmune disorders (4), such as atherosclerosis (5), obstructive pulmonary disease (6), rheumatoid arthritis, systemic lupus erythematosus, type 1 diabetes, and inflammatory bowel disease (7).

Bibliometrics is used to explore emerging trends in a specialized field (8), and the most commonly used bibliometric tools for its visualization are CiteSpace and VOSviewer, both of which are widely used in fields such as medicine, biology, and immunology (9, 10). Only few articles has revealed research trends in the field of efferocytosis (11, 12), but no bibliometric study has systematically characterized the relationship between efferocytosis and inflammation. Our study highlights the research hotspots and academic trends in this field for researcher with emphasis on the role of efferocytosis in the pathogenesis of inflammatory diseases and autoimmune diseases. We hope that these findings will provide new insights for future drug development and disease treatment.

## 2 Methods

### 2.1 Data collection

**Figure 1:**
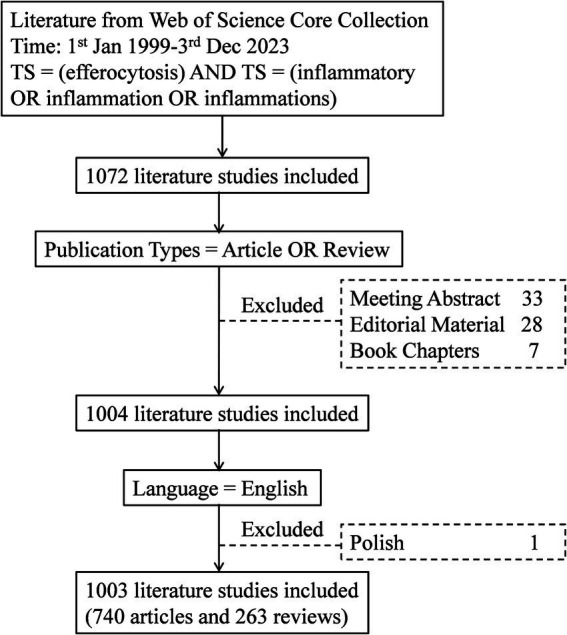
Flowchart of the literature selection process.

The data were obtained from the Web of Science Core Collection (WoSCC), with the following search formula: TS = (efferocytosis) AND TS = (inflammatory OR inflammation OR inflammations); the type was limited to treatises and reviews; the language was limited to English; and the search was conducted on December 03, 2023. The search process is shown in Figure 1. A total of 1,003 papers were included, of which 740 were treatises and 263 were reviews.

### 2.2 Data analysis

The retrieved literature data were imported into CiteSpace (version 6.2.R6) for further analysis (13). The exact parameters were set as follows: method (LLR), time slicing (January 2006–December 2023), year/slice(1), term source (title, abstract, author keywords, and keyword plus), and node type (keyword).

We evaluated the number of publications, major countries, research institutions, authors and keywords in the field of efferocytosis and inflammation research. We analyzed highly cited articles, and conducted co-occurrence analysis, clustering analysis and burst visualization of keywords. The keyword co-occurrence network consisted of nodes and connecting lines. The larger the nodes are, the more articles there are in that research direction, and the thicker the connecting lines are, the closer the association. Keyword clusters are network groups composed of keywords with similar research topics, reflecting the evolution of topics in the field over a certain time interval. The keyword timeline cluster graph introduces time into the network, presenting the historical trajectory and time span of the keyword evolution in each cluster. The results of the keyword bursts indicate a sharp increase in the intensity of a research direction over time and are used to identify research hotspots. Graphs were also generated via Microsoft Excel (version 2016), Scimago Graphica, Bibliometrix and R Studio.

## 3 Results

### 3.1 The global growth trend of publications

**Figure 2:**
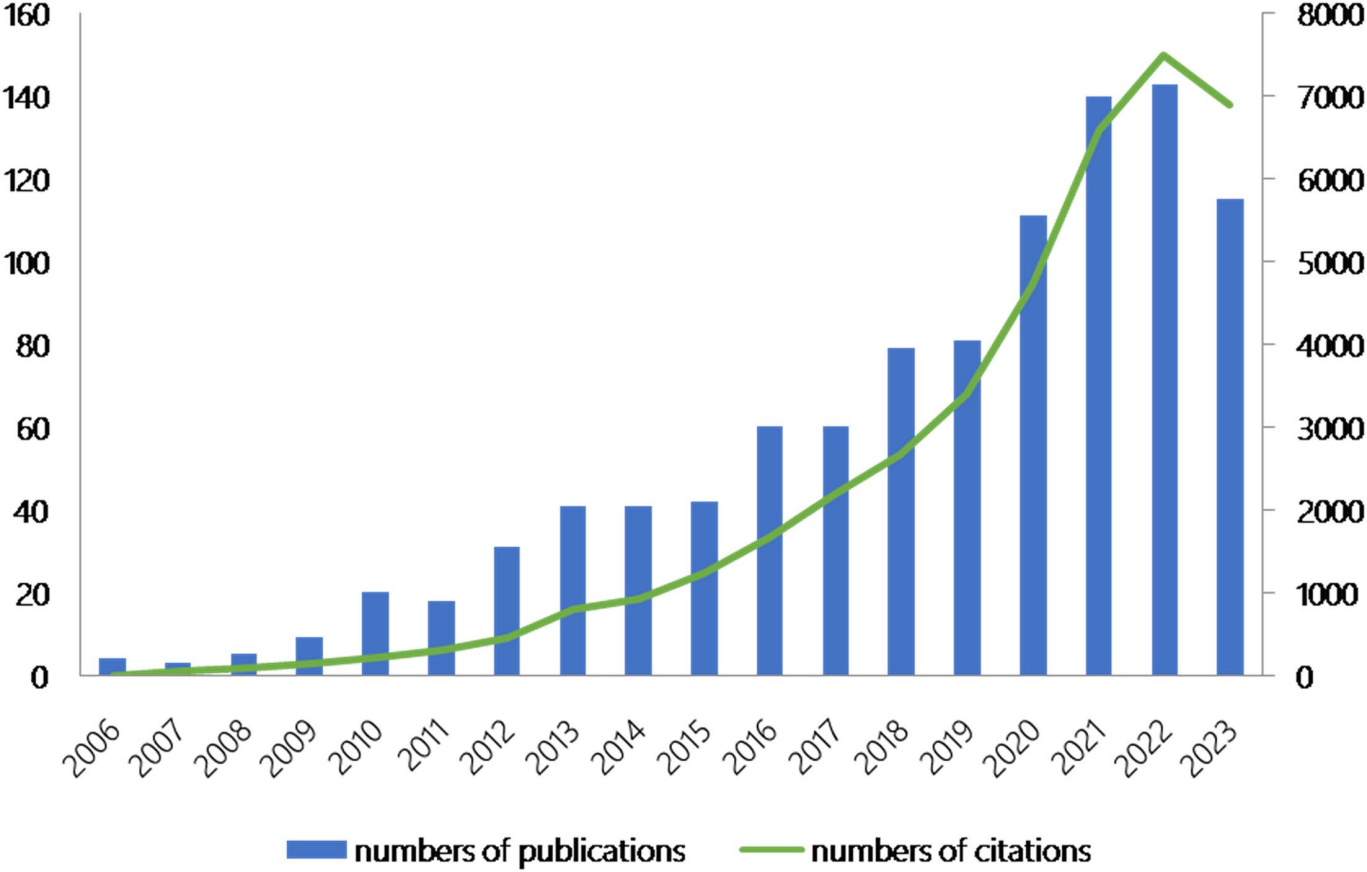
Publications and citations over time (2006–2023).

The number of publications is an essential indicator of the development trend of the research field. A total of 1,003 papers cited 45,109 publications, with an average of 39.86 citations and an h-index of 100 citations. Figure 2 shows that annual publications in the field rose from 4 in 2006 to 143 in 2022, and annual citations grew from 6 in 2006 to 7,496 in 2022. These studies spanned 74 research areas: “Immunology” (267, 26.62%) and “Cell Biology” (210, 20.94%) were published most frequently. Other popular research areas included “Biochemistry Molecular Biology” (156, 15.55%), “Pharmacology Pharmacy” (91, 9.07%) and “Medicine Research Experimental” (83, 8.28%).

### 3.2 Analysis of country contributions, institutions and authors

The United States ranked first in terms of the number of publications (456, 45.46%), followed by China (164, 16.35%) and the United Kingdom (99, 9.87%). Moreover, the majority of research collaborations centered between North America and Europe and between North America and East Asia (Supplementary Figure S1).

According to Supplementary Table S1, Harvard University was the institution that published the most papers (84, 6.74%). The 3 authors with the greatest number of publications were Serhan CN (35, 3.49%), Tabas I (29, 2.89%) and Dalli J (26, 2.59%). Among them, Serhan CN and Dalli J are more collaborative (14–16).

### 3.3 Analysis of journals and cocited journals

As shown in Supplementary Table S2, the largest number of publications (83, 8.28%) were from Frontiers in Immunology (7.3, Q1, from 2022), with a total of 2,545 citations. There were 14 articles published in Circulation Research (20.1, Q1, from 2022), with 1,287 citations. The favorite categories are Molecular/Biology/Immunology/Genetics journals (Supplementary Figure S2).

### 3.4 Analysis of research topics and frontiers

#### 3.4.1 Cluster network of the top 10 cited references and cocited references

Table 1 lists the top 10 most highly cited articles in the field of efferocytosis and inflammation. These highly cited articles suggest that scholars are interested in the association between efferocytosis and inflammation, with an emphasis on cell biological mechanisms and the associations with cardiovascular disease, lung disease and tissue repair.

**Table 1:** Top 10 cited references related to efferocytosis and inflammation.

Ranking	Title	Corresponding authors	Year	Journal	Citations	IF^a^	JCR-c
1	Embryonic and Adult-Derived Resident Cardiac Macrophages Are Maintained through Distinct Mechanisms at Steady State and during Inflammation	Epelman S	2014	Immunity	932	21.6	Q1
2	Inflammation and its resolution in atherosclerosis: mediators and therapeutic opportunities	Back M	2019	Nature Reviews Cardiology	729	20.3	Q1
3	Apoptosis and Clearance of Apoptotic Cells	Nagata S	2018	Annual Review of Immunology	530	21.4	Q1
4	Resolution of inflammation: an integrated view	Ortega-Gomez A	2013	Embo Molecular Medicine	499	8.2	Q1
5	Neutrophils orchestrate post-myocardial infarction healing by polarizing macrophages toward a reparative phenotype	Horckmans M	2017	European Heart Journal	442	23.4	Q1
6	Macrophage Dysfunction Impairs Resolution of Inflammation in the Wounds of Diabetic Mice	Khanna S	2010	Plos One	432	4.4	Q2
7	Specific lipid mediator signatures of human phagocytes: microparticles stimulate macrophage efferocytosis and pro-resolving mediators	Dalli J	2012	Blood	394	9.1	Q1
8	Macrophage proresolving mediator maresin 1 stimulates tissue regeneration and controls pain	Serhan CN	2012	Faseb Journal	345	5.7	Q1
9	Burying the dead—The impact of failed apoptotic cell removal (efferocytosis) on chronic inflammatory lung disease	Vandivier RW	2006	Chest	331	3.9	Q1
10	Efferocytosis in Health and Disease	Doran AC	2020	Nature Reviews Immunology	329	53.1	Q1

a IF (Impact Factor): All of the above impact factor was from the year the article published.

**Figure 3:**
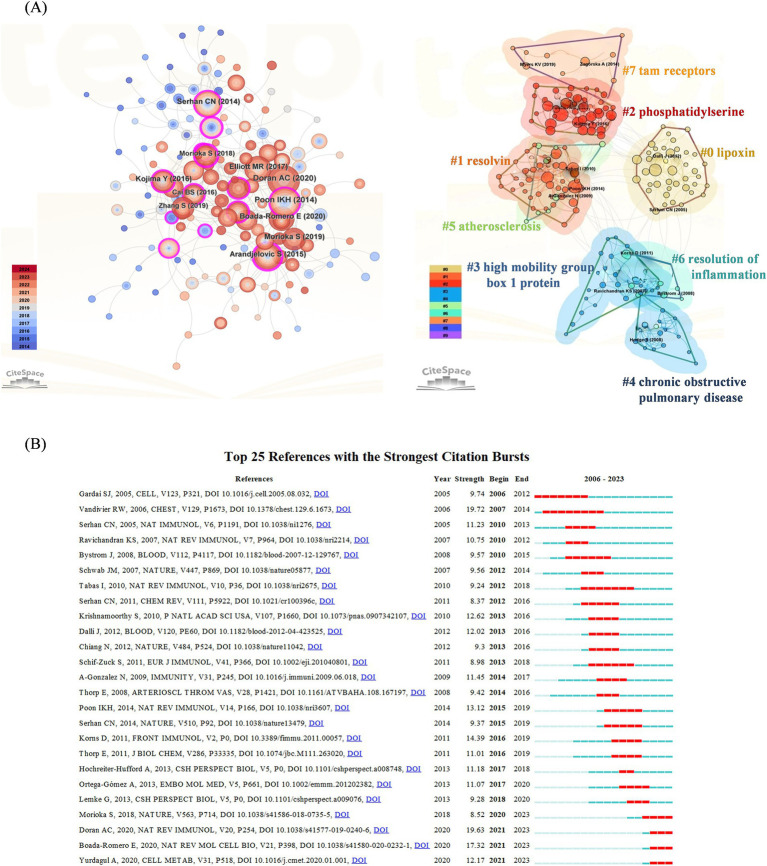
**(A)** Cocited references co-occurrence network and cluster analysis. The left subpanel visualizes the network of co-cited references, with node size indicating the citation frequency and node color representing the year of publication. The right subpanel highlights the major research themes identified through cluster analysis, with 8 clusters in total and distinguished by different colors. Cluster #0 is the largest. **(B)** Top 25 references with the strongest citation bursts. The blue line indicates the time-lapse, and the red line indicates the duration of the quote burst, which shows the progression of cutting-edge hot topics.

Cluster analysis of literature cocitations provides an objective reflection of the knowledge structure in the research area. Figure 3A shows that highly cited works, such as those by Poon et al. (72), are prominently displayed, indicating their significant influence in the field. Cluster #0 is the largest category, namely lipoxin, followed by resolving (Cluster #1), phosphatidylserine (Cluster #2), high mobility group box 1 protein (Cluster #3), chronic obstructive pulmonary disease (Cluster #4), atherosclerosis (Cluster #5), resolution of inflammation (Cluster #6) and Tam receptors (Cluster #7). These findings reveal that the research hotspots are focused mostly on proinflammatory mediators of efferocytosis and their conduction pathways, which is consistent with the research hotspots. Figure 3B shows the top 25 cocited references with strong bursts, and the first cocitation was initiated in 2006. It was published in *Cell* in October 2005 and was titled “Cell-surface calreticulin initiates clearance of viable or apoptotic cells through trans-activation of LRP on the phagocyte.” The strongest intensity of the burst was “Burying the dead—The impact of failed apoptotic cell removal (efferocytosis) on chronic inflammatory lung disease,” published in *Chest* in June 2006 by Vandivier RW. Overall, 4 publications describing the burst status were published in 2023, which suggests that future research on efferocytosis and inflammation will continue to evolve.

#### 3.4.2 Keywords co-occurrence

**Figure 4:**
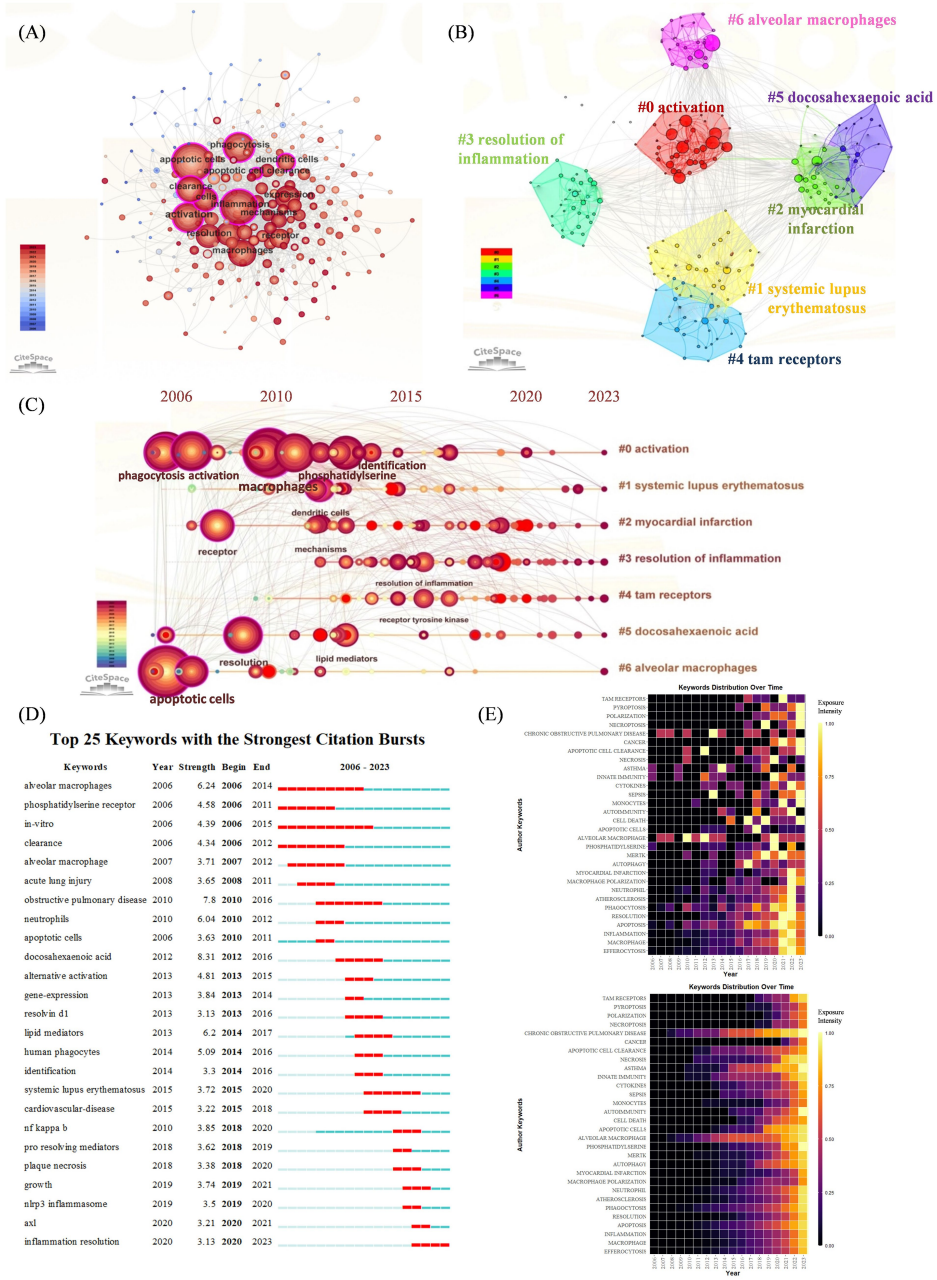
**(A)** Keywords co-occurrence network. The nodes represent keywords, and the larger nodes indicate more research articles. **(B)** Keyword cluster analysis. There are 7 clusters in total and are distinguished by different colors. Cluster #0 is the largest one. **(C)** Timeline view of keyword cluster from 2006 to 2023. The length of the horizontal straight line of a cluster indicates its time frame. Nodes and labels represent keywords that have been cited at least 45 times. **(D)** Top 25 keywords with the strongest citation bursts. The blue line indicates the time-lapse, and the red line indicates the duration of the quote burst, which shows the progression of cutting-edge hot topics. **(E)** Heatmap analysis of keywords. The intensity of the color in each box represents the level of attention a keyword received in a given year, with brighter colors indicating higher levels of attention. The upper heatmap illustrates the annual frequency of keyword bursts, highlighting the temporal distribution of keyword appearances. The lower heatmap depicts the cumulative keyword bursts, indicating the sequence and intensity of keywords gaining attention over the entire study period.

Co-occurrence refers to the occurrence of two or more keywords in the same article, thus, the keyword co-occurrence figure is plotted according to the frequency of keyword co-occurrence in the cited articles. As shown in Figure 4A, in addition to “efferocytosis” (105) and “inflammation” (237), the keywords with a co-occurrence frequency of more than 100 were “apoptotic cells” (261), “phagocytosis” (186), “activation” (155), “expression” (149), “clearance” (146), “macrophages” (142), “resolution” (130) and “receptor” (106).

#### 3.4.3 Analysis of keyword cluster timelines and keyword bursts

The results of the keyword cluster analysis are presented in Figure 4B, and a total of seven clusters were obtained. The largest cluster was Cluster #0, which was named “activation.” This was followed by “systemic lupus erythematosus,” “myocardial infarction,” “resolution of inflammation,” “tam receptors,” “docosahexaenoic acid,” and “alveolar macrophages.” A timeline plot of the keyword clusters is shown in Figure 4C. 2000s witnessed a surge of “activation” “myocardial infarction” and “alveolar macrophage,” introducing the identification of key receptors and signaling pathways involved in efferocytosis and inflammatory disease was a major advancement. After 2010, “systematic lupus erythematosus” and “resolution of inflammation” revealed that autoimmune disease and therapy has aroused researchers’ attention.

We further plotted keyword citation bursts via CiteSpace (Figure 4D), “docosahexaenoic acid” had the highest intensity (8.31), and those with longer citation burst durations included “alveolar macrophages” (2006–2014), “obstructive pulmonary disease” (2010–2016), and “systemic lupus erythematosus” (2015–2020). Many researchers have investigated these issues further. The most recent keyword used to describe the outbreak was “inflammation resolution” (2020–2023). The upper heatmap of Figure 4E is designed to show the frequency of a keyword’s burst during the years in detail, indicating the terms “chronic obstructive pulmonary disease,” “alveolar macrophages” had the longest citation durations. While the bottom heatmap shows a cumulative keyword burst, indicating the sequence of keywords gaining attention over the entire period, showing more recent outbreaks were associated with “cancer,” “pyroptosis,” “necroptosis” and “polarization.”

## 4 Discussion

### 4.1 General information

According to the WoSCC database, there was a trend toward an increase in the quantity of publications in this field, which occurred more rapidly from 2019 onward. This finding suggests that this area is gaining attention, which is consistent with previous findings (11).

It is noticeable that the U.S., especially the Harvard University, contributes most in the research field of efferocytosis and inflammation. The first landmark research from Harvard in the field of efferocytosis and inflammation can be traced back to the work on the role of phosphatidylserine and MerTK in apoptotic cell clearance published in 2001 by Scott et al. (17). This study was one of the first to elucidate the molecular mechanisms by which phagocytes recognize and engulf apoptotic cells, establishing a critical link between efferocytosis and immune regulation. In quick succession, Harvard’s researchers noticed that mice lacking the MerTK receptor exhibited delayed clearance of apoptotic cells and developed symptoms reminiscent of systemic lupus erythematosus (SLE) (18). This work highlighted the importance of efficient apoptotic cell clearance in preventing autoimmune responses and maintaining immune tolerance. Harvard’s contributions have been instrumental in defining the research landscape and guiding future investigations in this vital area of immunology.

Among the scholars in this field, Serhan CN has published the most articles and received the highest h-index, which reflects his outstanding contribution to the study of efferocytosis and inflammation. Notably, efferocytosis has been known for a long time in the academic field. However, it was not until 2003 that deCathelineau AM and colleagues named efferocytosis for the first time and elucidated the possible transmission pathway and mechanism of efferocytosis generation (19).

### 4.2 The role of efferocytosis in the pathogenesis of various inflammatory diseases and autoimmune diseases

A transition in research hotspots is depicted in Figures 4D,E, where the initial focus was on the role of efferocytosis in the pathophysiological processes of various inflammatory diseases (especially cardiac and pulmonary diseases), resulting in bursts of the keywords “*in vitro*,” “phosphatidylserine receptor” and “apoptotic cell clearance.” With the gradual improvement in the understanding of the underlying mechanisms, research hotspots have also progressively turned to treatment methods, so “inflammation resolution” has recently become a popular topic. Extensive studies of efferocytosis have been conducted in many inflammatory diseases, especially atherosclerosis, obstructive lung disease, systemic lupus erythematosus and rheumatoid arthritis.

**Figure 5:**
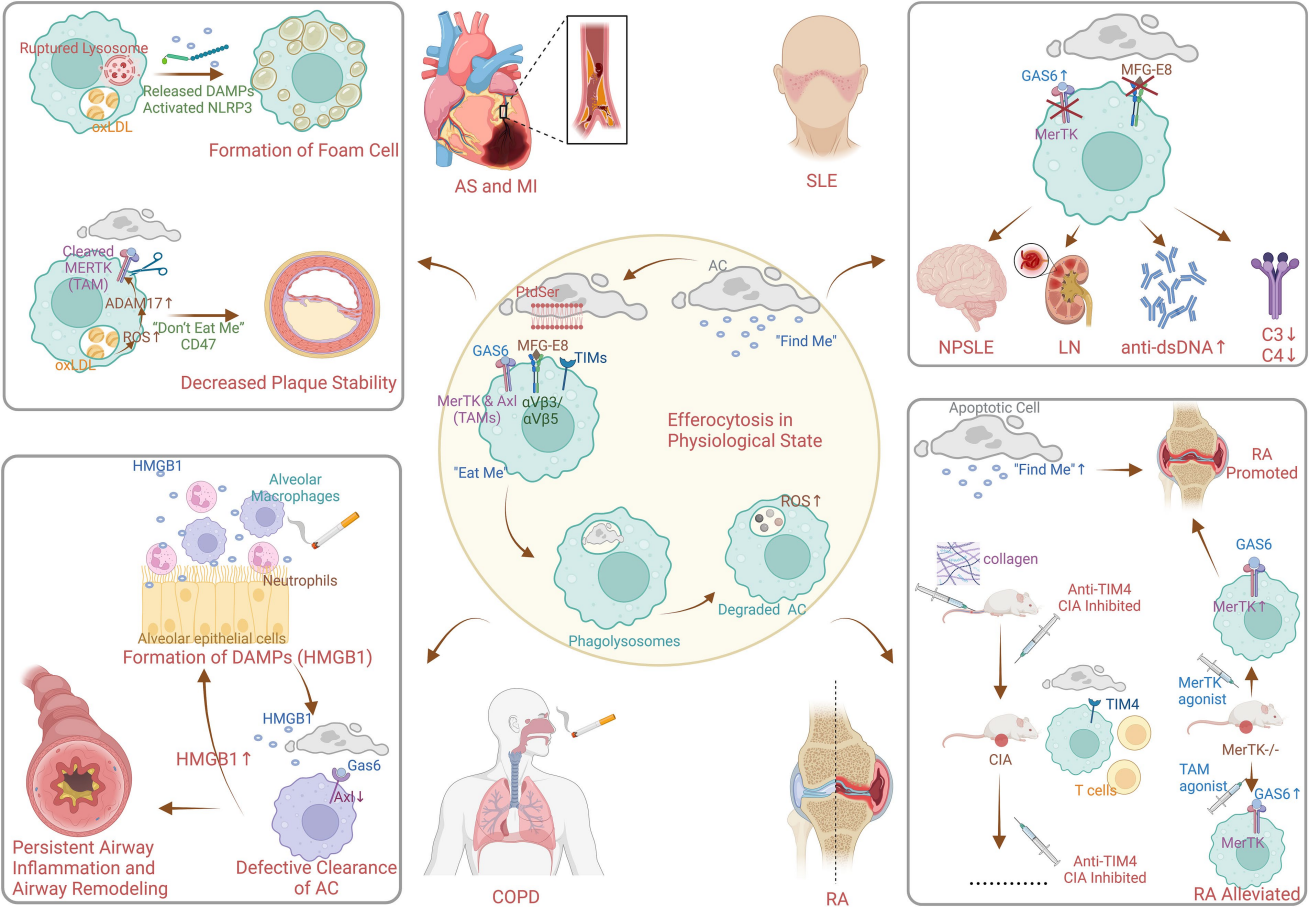
Mechanism of efferocytosis in physiological and pathophysiological state (created with BioRender.com). AC, apoptotic cell; PtdSer, phosphatedylserine; MFG-E8, milk fat globule EGF factor 8; GAS6, growth arrest specific protein 6; TIMs, T-cell immuneglobulin; MerTK, Mer protooncogene tyrosine kinase; Axl, anexelekto; TAMs, Tyro3/Axl/Mer receptor tyrosine kinases; ROS, reactive oxidative stress; DAMPs, damage-associated molecular patterns; NLRP3, nucleotide-binding oligomerization domain-like receptor protein 3; oxLDL, oxidized low-density lipoprotein; AS, atherosclerosis; MI, myocardial infarction; HMGB1, high mobility group box 1 protein; COPD, chronic obstructive pulmonary disease; SLE, systemic lupus erythematosus; NPSLE, neuropsychiatric systemic lupus erythematosus; LN, lunpus nephritis; RA, rheumatoid arthritis; CIA, collagen-induced arthritis.

The identification of key receptors and signaling pathways involved in efferocytosis was a major advancement in 2000s. In the process of efferocytosis, as shown in Figure 5, there are four stages: the identification of apoptotic cells (ACs), the binding of ACs, the internalization of ACs and the degradation of ACs (20). In the first phase, chemokines, known as “find me” signals, are triggered by ACs to induce the efficient mobilization of efferocytic immune cells. In the second phase, phagocytes, which are mediated by “find me” signals, accumulate abundant ACs, and phagocytosis receptors bind to “eat me” signals on the surface of ACs to initiate efferocytosis (21). Phosphatidylserine (PtdSer) is expressed on the surface of ACs and binds to the bridging molecules human growth arrest-specific protein 6 (GAS6) and milk fat globule-EGF factor 8 (MFG-E8). It is recognized by the Mer proto-oncogene tyrosine kinase (MERTK, a TAM receptor) and the integrin receptor αVβ3/αVβ5 on the surface of efferocytes (21). It can also directly bind to PtdSer receptors, such as T-cell immunoglobulin (TIM). During the third phase, the “eat me” signal binds to the receptor on the surface of phagocytes, mediating cytoskeleton reorganization and endocytosis of ACs to form phagolysosomes (22). Finally, lysosomes fuse with and acidify phagosomes to degrade internalized ACs, the key molecule of which is reactive oxygen species (ROS) (23).

Research in the 2010s highlighted the role of defective efferocytosis in the pathogenesis of chronic inflammatory diseases. Figure 5 also shows the pathophysiological process of efferocytosis in some non-neoplastic diseases. Lipid metabolism plays an essential role in the study of efferocytosis and atherosclerosis. In addition, macrophages consume oxidized low-density lipoprotein (oxLDL), which can lead to the formation of cholesterol crystals in lysosomes. This results in instability and even rupture of lysosomes and ultimately causes the release of unesterified cholesterol into the cytoplasm, NLRP3 inflammasome activation, and foam cell formation (24–26). In addition, ROS promote the activation of the protease ADAM17 on macrophages, which, by cleaving MERTK (27), causes ACs to inappropriately express the “do not eat me’ signal CD47. This leads to impaired clearance of ACs from atherosclerotic plaques, increased area of plaque lipid necrotic nuclei, and decreased plaque stability (28). The reduction in inflammation is mediated by specialized proresolving lipid mediators from omega-3 fatty acids or arachidonic acid, as well as associated proteins and signaling gas molecules; this pathway reduces inflammatory vesicle formation, alleviates oxidative stress, and enhances efferocytosis (29). Moreover, enhancing efferocytosis in atherosclerotic lesions through CD36 and MERTK receptor activation has been proposed as a strategy to reduce plaque burden and stabilize plaques, potentially decreasing the risk of cardiovascular events (3).

Existing studies on the pathogenesis of efferocytosis in obstructive pulmonary disease have focused on multiple fields. First, high mobility group box 1 protein is a typical damage-associated molecular pattern (DAMP) protein that is secreted by airway cells exposed to cigarettes and other substances, such as neutrophils, alveolar macrophages, lymphocytes, and epithelial cells (30). It binds to receptor for advanced glycosylation end products (RAGE) and Toll-like receptor 4 (TLR4) to exert its activity (31, 32), resulting in the nuclear activation and translocation of NF-κB (33). This triggers airway inflammation and plays an influential role in airway remodeling due to persistent airway inflammation. Second, Hodge S et al. verified that dysregulation of PtdSer-recognizing receptors or bridging molecules may be responsible for defective clearance of ACs in COPD (34). However, relatively few studies on PtdSer-recognizing receptors or bridging molecules exist, and only a limited number of studies have suggested that MerTK upregulation is not sufficient to normalize macrophage efferocytosis (35, 36). In fact, animal studies have shown that another TAM receptor “Axl,” expressed in mouse airway macrophages, is effectively expressed under stimulation by inflammatory factors such as type I interferon or Toll-like receptor-3 stimulation and binds to Gas6 (37).

Systemic lupus erythematosus (SLE) is an autoimmune disease characterized by overactivation of immune cells and overproduction of autoantibodies, resulting in systemic involvement of multiple organs. In SLE, efferocytosis is often defective. Inefficient clearance of apoptotic cells can lead to the accumulation of cellular debris, which can trigger an autoimmune response. This debris can be recognized as foreign by the immune system, leading to the production of autoantibodies and the formation of immune complexes (3). The efferocytosis bridging molecules described previously play an active role in the pathogenesis of SLE. Several studies have demonstrated that the serum level of the efferocytosis bridging molecule Gas6 is significantly elevated in SLE patients, which is associated with neurological involvement, plasmacytosis, renal dysfunction, high dsDNA antibody titers, and decreased levels of complement C3 and C4 (38), which is probably due to the targeting of the MerTK (17). Therapies that upregulate MerTK on macrophages have shown promise in preclinical models of SLE (18). In addition, the results of *in vitro* experiments indicated that a lack or excess of another efferocytosis bridging molecule, MFG-E8, impeded efferocytosis (39). Hanayama R. demonstrated that MFG-E8-deficient mice accumulate ACs in germinal centers and spontaneously produce autoantibodies to develop lupus-like autoimmune disease (40). Moreover, high levels of MFG-E8 have been detected in sera from human SLE patients (41).

Rheumatoid arthritis is a chronic synovial inflammatory disease that progressively contributes to cartilage and bone destruction and the risk of disability. It has been shown that efferocytosis “find me” signaling chemokines CX3CL1 (42), ATP (43), and sphingosine-1-phosphate (44) promote rather than attenuate the pathophysiological processes of RA (45–49). Interestingly, during the binding phase of efferocytosis to ACs, the induction phase of collagen-induced arthritis (CIA) is neutralized by the direct PtdSer receptor “T-cell immunoglobulin and mucin structural domain 4 (TIM4),” which exacerbates inflammation in joints. Abe Y suggested that this effect was possibly due to the effect of TIM4 on the development of T-cells (50). In contrast, treatment with anti-TIM4 administered before or after the onset of CIA significantly inhibited the development and progression of CIA by reducing proinflammatory cytokines without affecting T or B-cell responses, suggesting that anti-TIM4 treatment may be a suitable target for the treatment of RA (50). MerTK is a member of the TAM family of cytosolic indirect receptors, and MerTK^−/−^ mice presented increased joint inflammation (51), which was attenuated by the overexpression of the TAM receptor agonist Gas6 and protein S (52). However, mice treated with MerTK-specific agonist antibodies also unexpectedly exhibited exacerbated joint inflammation, which was suggested by Waterborg CEJ to be related to increased numbers of efferocytosis ACs in the knee joint and elevated serum interleukin-16C levels (51). Inhibition of integrin αVβ3 attenuated joint inflammation in arthritic rabbits and rats, although this effect may be independent of the indirect efferocytosis receptor MFG-E8 (53).

In addition to systemic lupus erythematosus and rheumatoid arthritis, efferocytosis has received increasing attention in the study of autoimmune diseases involving type 1 diabetes mellitus, inflammatory bowel disease, and multiple sclerosis (MS), where the mechanism may involve defective clearance of dead cells associated with an intolerant immunogenic response and dendritic cell maturation in chronic inflammation. A study on nonobese mice that instinctively progressed to T1DM demonstrated defects in efferocytosis mechanisms underlying the development of ANA both *in vivo* and *in vitro* (54). Lipopolysaccharide-binding proteins, Toll-like receptor 4, and bacterial permeability-increasing proteins have been detected in the serum of patients with ulcerative colitis, and these complexes are recognized by CD14. CD14 is linked to ICAM3 and promotes the recognition and phagocytosis of ACs (55). MS is a degenerative disease of the central nervous system characterized by focal lesions with inflammation, oligodendrocyte death, demyelination, and axonal damage. ATP is a major neurotransmitter in the central nervous system that activates ionotropic (P2X) and metabotropic (P2Y2) receptors, which both recognize different “eat me” and “find me” signals during cytosolic drinking (43, 56). Therefore, P2X and P2Y can be considered possible targets of MS. However, how defective clearance of apoptotic nerve cells contributes to the pathogenesis of MS is unclear. The above efferocytosis molecular pathways in autoimmune diseases are poorly defined and understudied and will be the focus of future developments in the field of efferocytosis and inflammation.

### 4.3 Research frontiers and future prospects

Emerging surge keywords like “cancer” and “inflammation resolution” forebode research frontiers. Efferocytosis, the process by which phagocytes clear apoptotic cells, has a controversial and paradoxical role in cancer. On one hand, efferocytosis can promote anti-tumor immunity by efficiently clearing apoptotic cancer cells, thereby preventing secondary necrosis and the release of pro-inflammatory and potentially immunogenic cell contents. This clearance helps maintain tissue homeostasis and can facilitate the recruitment and activation of immune cells that target tumor cells (57). On the other hand, efferocytosis can also contribute to tumor progression by creating an immunosuppressive environment. The ingestion of apoptotic cells by macrophages and other phagocytes can lead to the release of anti-inflammatory cytokines, such as TGF-*β* and IL-10, which suppress effective anti-tumor immune responses and promote tumor growth and metastasis (58). This dual role complicates the development of therapeutic strategies that aim to modulate efferocytosis in cancer, as enhancing efferocytosis might inadvertently support tumor progression in certain contexts (59).

While efferocytosis presents a complex dual role in cancer, its potential in resolving inflammation highlights a promising avenue for therapeutic exploration, particularly with the use of mesenchymal stem cells (MSCs). Inflammation resolution has become a research trend in recent years. MSCs have been shown to have an important research value and broad translational application prospects. Previous studies have shown that the efferocytosis of MSCs can be used to alleviate lung (60) and synovial (61) inflammation, improve myocardial ischemia/reperfusion (62), and enhance the therapeutic effect of sepsis (63) based on the mechanism of the shift of macrophages from a proinflammatory phenotype to an inflammation-suppressive phenotype through releasing anti-inflammatory cytokines. In turn, a therapeutic modality derived from these MSC exosomes or vesicles has been shown to alleviate lupus nephritis (64), prevent complications after vascular stent insertion (65), and ameliorate insulin resistance in type 2 diabetes mellitus patients (66). Although MSC shows inconspicuous effects in limited preclinical researches of autoimmune liver disease and Crohn’s disease (67), clinical application of stem cells still facing numerous obstacles to the application of stem cell therapy at this stage because of insufficient research. Safety concerns, including the risk of tumorigenesis and immune rejection, remain significant hurdles (68). The variability in efficacy depending on stem cell source, delivery method, and timing of administration necessitates further optimization (69). Additionally, regulatory and ethical considerations complicate the approval and implementation of stem cell therapies (70). Finally, the high cost of stem cell treatments poses a barrier to widespread clinical adoption, necessitating efforts to improve cost-effectiveness and accessibility (71). Addressing these challenges through rigorous research and collaboration among scientists, clinicians, and regulatory bodies is essential for harnessing the full therapeutic potential of stem cells in efferocytosis and inflammatory diseases.

However, there is significant redundancy in the mechanisms that regulate efferocytosis. When one pathway is inhibited, others may compensate, making it challenging to develop targeted therapies. Developing therapies that modulate efferocytosis without unintended side effects is challenging. Enhancing or inhibiting efferocytosis could have unpredictable consequences depending on the disease context.

### 4.4 Limitations

Some limitations inherent to bibliometrics are present in our study. First, the data were extracted only from the WoSCC database, possibly missing some important findings published in other databases. Nonetheless, the WoSCC is a definitive and comprehensive database in the field of medicine. The amount of data we analyzed was large enough to reflect research in the field of efferocytosis and inflammation. Multiple databases are recommended to be searched in the future work. Moreover, VOSviewer and CiteSpace may have missed some information due to the inability to analyze the full texts of the publications, causing bias in other bibliometric studies. A more impartial software in bibliometric analysis is expected to be created and used.

## 5 Conclusion

Bibliometric analysis provides an objective and quantitative method for evaluating research directions toward efferocytosis and inflammation. Recent research shows that efferocytosis plays an important role in the pathogenesis of a variety of inflammatory and autoimmune diseases such as atherosclerosis, obstructive pulmonary disease, SLE and RA. Inflammation resolution by efferocytosis may be a potential method for treating not only inflammatory diseases but also cancer. However, the feasibility of targeting efferocytosis for the treatment of diseases requires further in-depth research.

## Data Availability

The original contributions presented in the study are included in the article/Supplementary material, further inquiries can be directed to the corresponding authors.
