# Supplementary material for: Efferocytosis and inflammation: a bibliometric and systematic analysis
Source: Front Med (Lausanne). 2025 Feb 10;12:1498503. doi: 10.3389/fmed.2025.1498503 (PMC11847848; doi:10.3389/fmed.2025.1498503)
Supplement: Supplementary file 1 [file Data_Sheet_1.doc]

Supplementary Table 1 Top 5 based on the number of publications

| Field |  | Record Count | % of 1003 |
| --- | --- | --- | --- |
| Research areas | Immunology | 267 | 26.62 |
|  | Cell Biology | 210 | 20.94 |
|  | Biochemistry Molecular Biology | 156 | 15.55 |
|  | Pharmacology Pharmacy | 91 | 9.07 |
|  | Medicine Research Experimental | 83 | 8.28 |
| Affiliations | Harvard University | 84 | 6.74 |
|  | Institut National de la Sante et de la Recherche Medicale | 58 | 4.65 |
|  | University of London | 39 | 3.13 |
|  | Columbia University | 36 | 3.59 |
|  | Queen Mary University of London | 31 | 2.49 |
| Authors | Serhan CN | 35 | 3.49 |
|  | Tabas I | 29 | 2.89 |
|  | Dalli J | 26 | 2.59 |
|  | Teixeira MM | 17 | 1.69 |
|  | Sousa LP | 16 | 1.60 |

Supplementary Table 2 Top 10 cited and cocited journals in efferocytosis and inflammation field

| Ranking | Journals | Articles | Citations | IF* | JCR | Cocited journals | Co-citations | IF* | JCR |
| --- | --- | --- | --- | --- | --- | --- | --- | --- | --- |
| 1 | Frontiers in Immunology | 83 | 2545 | 7.3 | Q1 | Journal of Immunology | 3981 | 4.4 | Q2 |
| 2 | Journal of Immunology | 34 | 2139 | 4.4 | Q2 | Nature | 2420 | 64.8 | Q1 |
| 3 | Plos One | 25 | 1386 | 3.7 | Q2 | Journal of biological chemistry | 2148 | 4.8 | Q2 |
| 4 | Cells | 24 | 190 | 6 | Q2 | Journal of clinical investigation | 1960 | 15.9 | Q1 |
| 5 | Cell Death & Disease | 19 | 561 | 9 | Q1 | Proceedings of the National Academy of Sciences | 1931 | 11.1 | Q1 |
| 6 | Journal of Leukocyte Biology | 18 | 578 | 5.5 | Q2 | Journal of experimental medicine | 1556 | 15.3 | Q1 |
| 7 | Faseb Journal | 15 | 945 | 4.8 | Q1 | Blood | 1510 | 20.3 | Q1 |
| 8 | Circulation Research | 14 | 1287 | 20.1 | Q1 | Frontiers in Immunology | 1493 | 7.3 | Q1 |
| 9 | Arteriosclerosis Thrombosis and Vascular Biology | 14 | 635 | 8.7 | Q1 | Nature Reviews Immunology | 1408 | 100.3 | Q1 |
| 10 | Scientific Reports | 14 | 396 | 4.6 | Q2 | Cell | 1366 | 64.5 | Q1 |

* IF (Impact Factor): all of the above cited impact factor was from 2022.

Supplementary Figure 1 A world map describing contributions and co-operations of countries.

The size of the circles represents the volume of articles, with larger circles indicating a higher number of publications. The connecting lines depict collaborative efforts between countries, with thicker lines signifying stronger collaborative links.

Supplementary Figure 2 The dual-map overlay of journals.

The left side of the figure represents the citing journals, while the right side depicts the cited journals. Colored lines link the two sides, illustrating citation pathways and showing how research in various fields is interconnected.
