# Supplementary figures and images for: Efferocytosis and inflammation: a bibliometric and systematic analysis
Source: Front Med (Lausanne). 2025 Feb 10;12:1498503. doi: 10.3389/fmed.2025.1498503 (PMC11847848; doi:10.3389/fmed.2025.1498503)

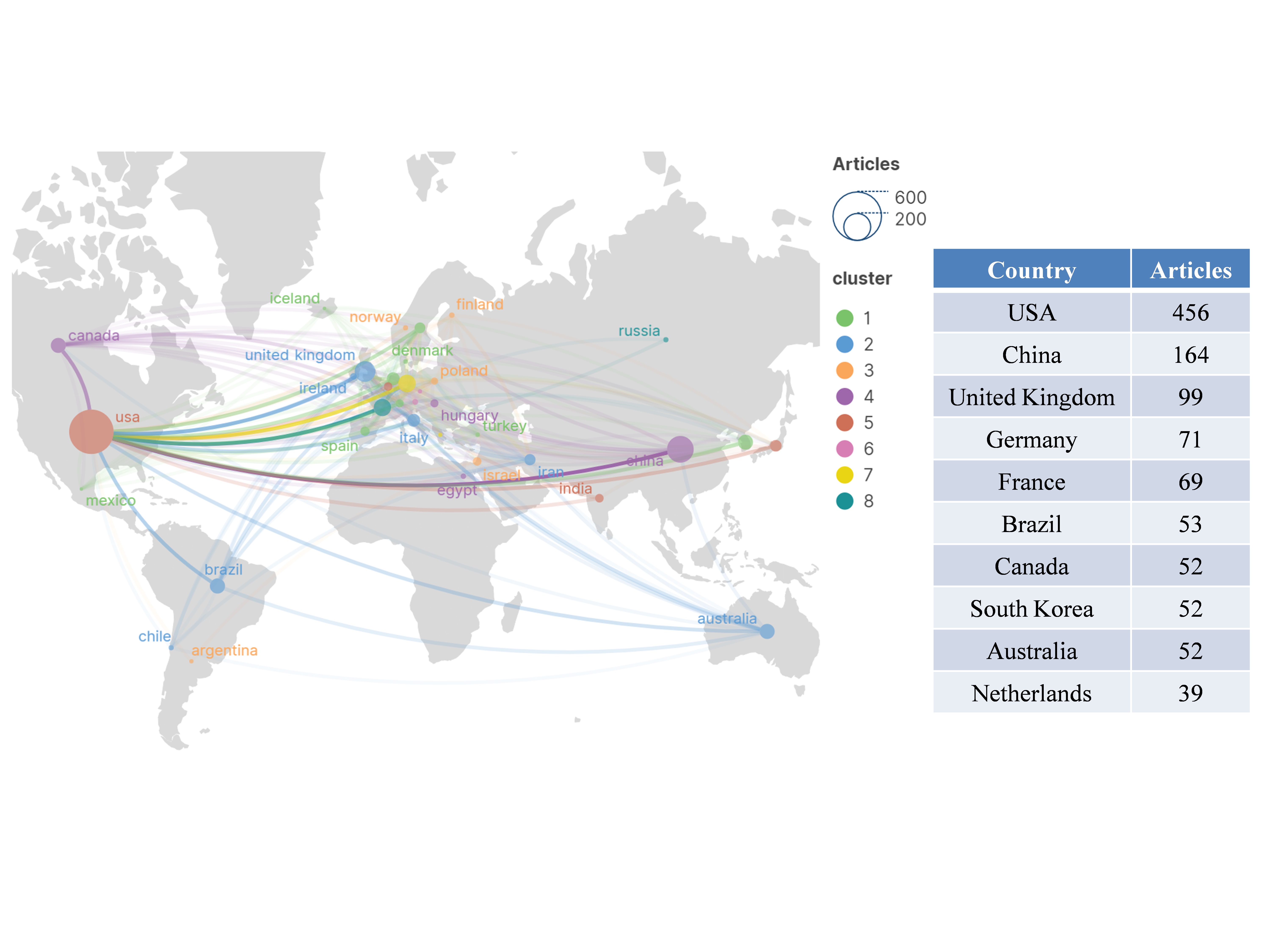

Supplement: Supplementary file 2 [file Image_1.jpeg]

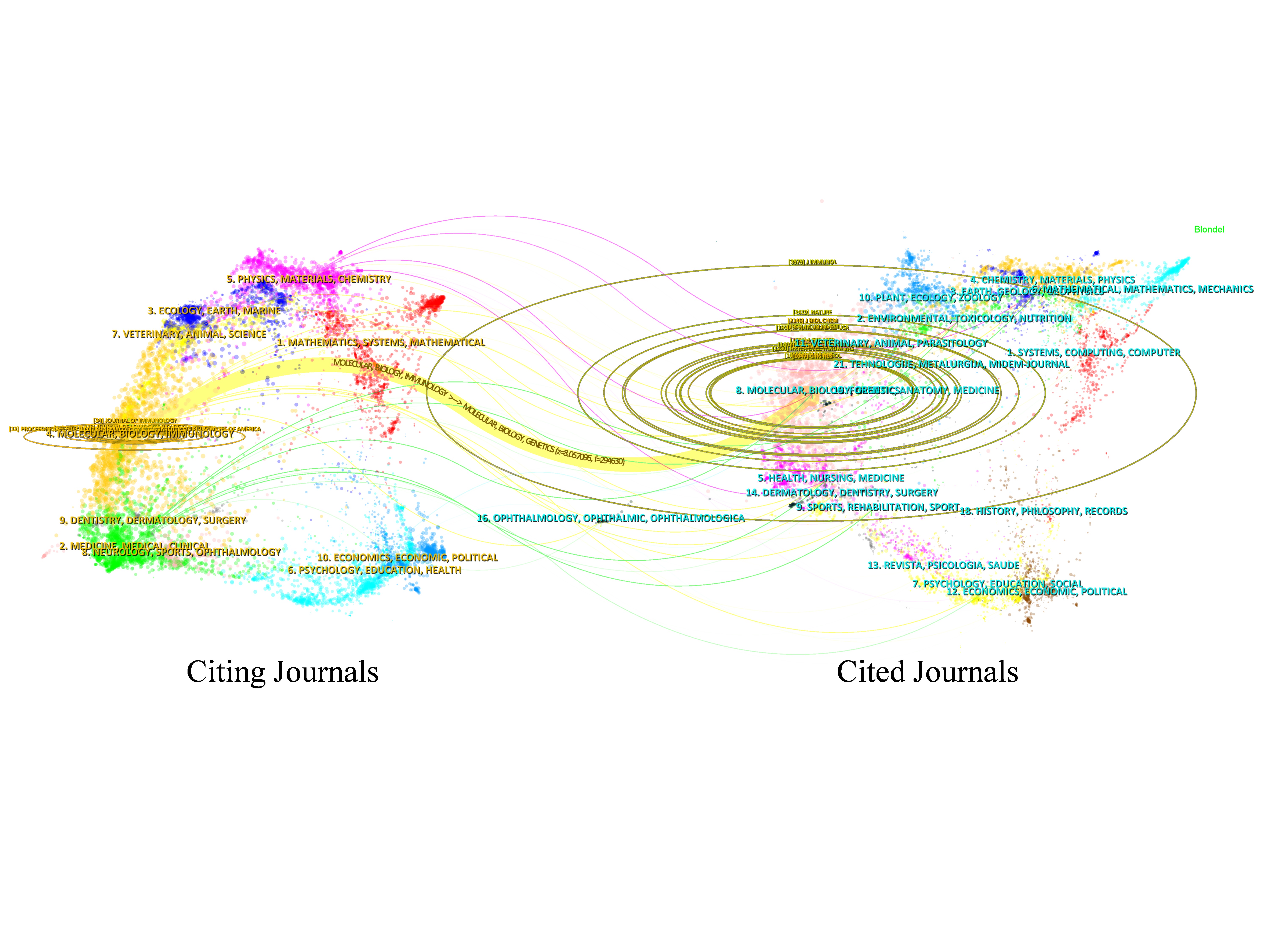

Supplement: Supplementary file 3 [file Image_2.jpeg]
